# Testing the Applicability of MALDI-TOF MS as an Alternative Stock Identification Method in a Cryptic Species Complex

**DOI:** 10.3390/molecules25143214

**Published:** 2020-07-14

**Authors:** Gabor Maasz, Zita Zrínyi, Istvan Fodor, Nóra Boross, Zoltán Vitál, Dóra Ildikó Kánainé Sipos, Balázs Kovács, Szilvia Melegh, Péter Takács

**Affiliations:** 1 Balaton Limnological Institute, Centre for Ecological Research, Klebelsberg Kuno street 3, 8237 Tihany, Hungary; zrinyi.zita@okologia.mta.hu (Z.Z.); fodor.istvan@okologia.mta.hu (I.F.); boross.nora@okologia.mta.hu (N.B.); vital.zoltan@okologia.mta.hu (Z.V.); takacs.peter@okologia.mta.hu (P.T.); 2 Department of Aquaculture, Szent István University, Páter Károly str. 1, 2100 Gödöllő, Hungary; kanaine.sipos.dora.ildiko@mkk.szie.hu (D.I.K.S.); Kovacs.Balazs@mkk.szie.hu (B.K.); 3 Department of Medical Microbiology and Immunology, Medical School, University of Pécs, Szigeti str. 12, 7624 Pécs, Hungary; melegh.szilvia@yahoo.co.uk

**Keywords:** phyloproteomics, MALDI-TOF mass spectrometry, gobio, freshwater fish, classification, taxonomy

## Abstract

Knowledge of intraspecific variability of a certain species is essential for their long-term survival and for the development of conservation plans. Nowadays, molecular/genetic methods are the most frequently used for this purpose. Although, the Matrix Assisted Laser Desorption Ionization Time of Flight Mass Spectrometry (MALDI-TOF MS) technique has become a promising alternative tool to specify intraspecific variability, there is a lack of information about the limitations of this method, and some methodological issues need to be resolved. Towards this goal, we tested the sensitivity of this method on an intraspecific level, using genetically identified individuals of a cryptic fish species complex collected from five distinct populations. Additionally, some methodologic issues, such as the effect of (1) delayed sample preparation, (2) clove oil anaesthetization, and (3) different tissue types (muscle, and brain) were investigated using the MS analysis results. Our results show that the delayed sample preparation has a fundamental effect on the result of MS analysis, while at the same time the clove oil did not affect the results considerably. Both the brain and muscle samples were usable for cryptic species identification, but in our opinion this method has limited applicability for population-level segregation. The application of MALDI-TOF MS to the exploitable toolkit of phylogenetic and taxonomic researches could be used to broaden conclusions.

## 1. Introduction

The severe habitat degradation and mass species extinctions are the most obvious evidence of the global biodiversity crisis [1,2]. At the same time, there are several less spectacular, but just as important signs of degradation, which are much more difficult to quantify. Among others, the decline of the intraspecific variability of a certain species has a specific negative effect on its long term survival. Therefore, this information is essential in conservation planning and in preserving the natural community structure [3].

It has long been suspected that many species, notably those spread over a wide geographic range, can be divided into numerous more or less discrete, but phenotypically/morphologically very similar entities, the so-called cryptic or sibling species [4]. The widespread application of molecular techniques has proven that these entities can be found in many animal groups [5]. The importance of these cryptic entities is still insufficiently considered, despite the fact that they are required for wildlife and biodiversity conservation, as well as natural resource protection [6,7]. Therefore, their identification and description are critically important. Moreover, these reproductively more or less isolated groups are the basic units of evolutionary development [8].

As the considerable proportion of intraspecific diversity of freshwater fish species is manifested rather in among groups, than in within groups differences/variability [9], the preservation of local forms, subspecies, geographically isolated assemblages have particular importance in this species group.

In the last decades, molecular and genetic methods have become the fundamental tools of phylogenetics and taxonomy. These methods are widely used for species identification and intraspecific studies (e.g., population genetics) [10,11,12,13]. At the same time, these molecular methods are costly and time consuming, and still have substantial consumable requirements [14,15,16]. Moreover, these methods also require highly skilled laboratory staff. For these reasons, it is worthwhile to examine the applicability of newly developed methods for the detection of intraspecific variability. For example Matrix Assisted Laser Desorption Ionization-Time of Flight Mass Spectrometry (MALDI-TOF MS) is an equally reproducible, accurate, fast, and affordable candidate to investigate this feature [17,18,19].

Mass spectrometry based phyloproteomics (MSPP) has also been considered as an appropriate, user-friendly species identification tool for the interpretation of information encoded in genomes, complementing DNA-based approaches [20]. Among these proteomic tools, the MALDI-TOF MS is a well-established technique for the identification of specific marker compounds and proteomic phenotyping, due to its high sensitivity, high throughput and relatively low additional cost [21]. The first widely used application of this method was the bacterial identification for clinical microbiology [22,23,24,25,26,27,28,29], and has been recently adapted to food analysis and authentication [30,31,32]. Many examples show that this method can be used, not only in clinical trials, but in conservation biology research. Occasionally, it has been used to identify microalgae [19,33] and even higher eukaryotes, including nematodes [34], insects [35,36,37], molluscs [18,38], and fish [39,40]. Moreover, the results of specific studies show that it can also be used for differentiating closely related, morphologically very similar species [16,41], and for the identification of proteomic sex markers in fishes and in arthropods [15,42].

Although, this method has rarely been previously tested at intraspecific levels (e.g., for population detachments) [43,44], the results of these studies show that its sensitivity may make it suitable for stock identification as well.

There are also many uncertainties and elusive details in the sampling methodology (field sampling, sample preparation and processing, etc.) for MALDI-TOF MS purposes. However, these features may also fundamentally affect the results of these kind of studies [45,46].

Therefore, our aims were to elucidate the applicability and sensitivity of an alternative (rapid MSPP) method for the determination of intraspecific diversity, in order to differentiate cryptic species and populations - in a freshwater Cyprinid superspecies complex. Parallel genetic and MALDI-TOF MS investigations were executed on the same stream-dwelling gudgeon (*Gobio* spp.) individuals collected from five distinct populations. We also wanted to clarify certain methodological issues of the MALDI-TOF MS investigations by conducting these investigations in parallel. Therefore, two different tissue types (brain and muscle) were analyzed to test their suitability for this purpose. Moreover, three types of field sample processing protocols were compared to reveal the effects of anaesthesia and middle term storage on the results of mass spectral analysis.

## 2. Results

### 2.1. Phylogenetic Differences of the Studied Individuals

**Figure 1:**
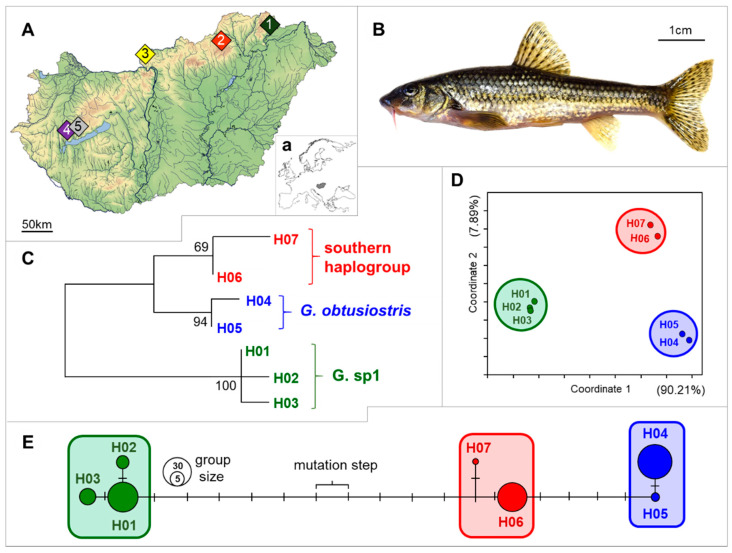
Geographic distribution of sample sites in Hungary (**A**) and the location of Hungary in Europe (**a**); physical appearance of a studied *Gobio* specimen (**B**). Maximum likelihood tree showing the divergence of the seven haplotypes derived from the 608 bp long mtCR sequence data of the studied gudgeon individuals. The posterior probabilities of divergence are shown next to the branches (**C**). PCoA plot derived from the pairwise nucleotide differences of haplotypes. The attributed variance in each axis is indicated in parenthesis (**D**). Median-Joining network of mtCR sequence data. Circle sizes are relative to the number of individuals carrying the same haplotype. Line length refers to the genetic distances of haplotypes. Color codes and enframings are identical for all subfigures. For more details see Table 1 (**E**).

Aligned sequences of 608 3′- ends of the mitochondrial control region (mtCR) were obtained from the 90 individuals studied, which were grouped into seven haplotypes (Table 1). The results of GenBank query showed that the indicated sequences (H01-07) were identical with the ones owning the following GenBank accession numbers: KC757339, KC757341, KC757342, KC757328, KC757329, KC757330, KC757332, respectively. Although the net nucleotide differences varied between haplotypes from 1 to 13 (0.16–2.14%), three different analysis methods including Maximum Likelihood tree, Principal Coordinate Analysis (PCoA) plot derived from the pairwise nucleotide differences of haplotypes, and the Median-Joining network of mtCR sequence data showed that the seven haplotypes can be classified into three distinct haplogroups (Figure 1C–E). The mean nucleotide difference within and between groups was 1.6 ± 0.9 SD (0.26 ± 0.15% SD) and 10.25 ± 2.7 SD (1.69 ± 0.46% SD), respectively. Moreover, these three groups-H01-02-03, H04-05, and H06-07- are identical with those that were mentioned as “G. sp. 1”, “*G. obtusirostris*” and “Southern haplogroup” in our previous publication [47]. From these three groups the *G. obtusirostris* Valenciennes, 1842 is the only valid species. From the other two groups the Southern haplogroup shows higher similarity to the *G. obtusirostris* than the G. sp.1.

### 2.2. Effect of Different Sample Preparation Methods on MALDI-TOF MS Results

**Figure 2:**
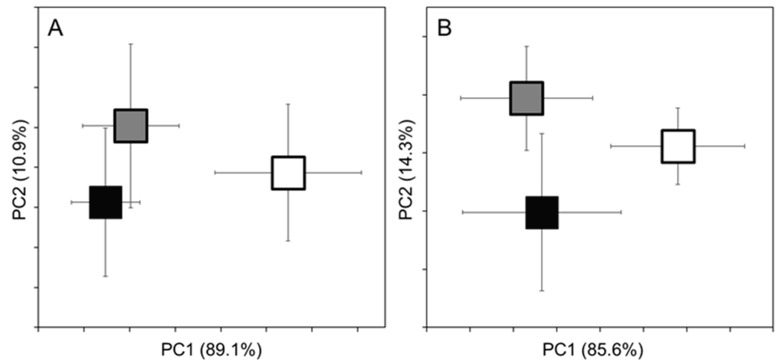
Principal Component Analysis plots of MALDI-TOF MS spectral datasets derived from gudgeon brain (**A**) and muscle (**B**) tissues. The groups were assigned based on the sample preparation type independently from the collection site and genetic features. Black square (Prep. Type 1): immediate field preparation; Grey square (Prep. Type 2): clove oil euthanization + immediate field preparation; White square (Prep. Type 3): the whole body was frozen and prepared in the lab after 30 days incubation on −30 °C. For clarity, only the group centroids are shown, with vertical and horizontal error bars indicating the standard deviation of data. The explained variance in each axis is indicated in parenthesis. The original PCA showing all individuals data are available in the Supplementary Figure S1).

During the methodological survey of brain and muscle tissue, 90 and 92 MS peaks were detected, and the detected peaks ranged between 4124.079 and 20,337.387 Da and 4912.896 and 13,453.94 Da, respectively (Table S1). Results of the Principal Component Analysis (PCA) analysis of both brain and muscle mass spectra data show that the on-site prepared samples (preparation types 1 and 2) are separated from those were processed in the lab after 30 days incubation at −30 °C degrees (preparation type 3). The separation was significant along the PC1 axis–which explains the majority of variance–determined by the results of a Kruskall-Wallis (KW) non-parametric test (*p* < 0.01) in both cases (for the pairwise values see Table 2). At the same time, preparation type 1 and 2 showed considerable overlap, and show slight separation on the PC2 axis (Figure 2).

The classification shows similar results as the PCA plots. The MALDI-TOF MS method was able to classify 59.6% and 66.6% of the cases correctly into the three preparation method type in the case of brain and muscle samples, respectively. These relatively low values can be attributed to the MALDI MS spectral analysis not reliably separating the individuals prepared by the first and second methods. On the contrary, the third preparation method showed considerably higher percentage of correctly classified cases (CCC). Moreover, all muscle samples prepared by the third methodology were correctly classified (Table 3). Although we found some peaks to be specific for the certain sample preparation types, but none of these peaks appeared in the spectra of the all individuals classified into the certain group. At the same time several peaks were more frequent in the samples prepared after 30 days incubation (For more details see Table S1).

### 2.3. Cryptic Species Identification

**Figure 3:**
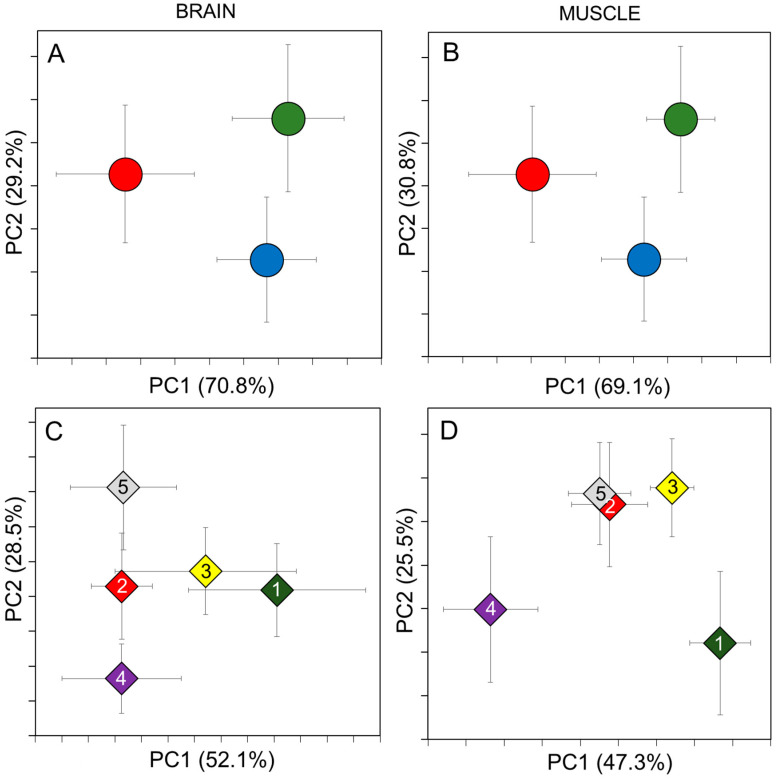
Principal Component Analysis plots of MALDI-TOF MS data derived from brain and muscle samples. Groups were set up by genetic features (**A**,**B**) and collection site (**C**,**D**). Red circle: Southern haplogroup, Blue: *G. obtusirostris*, Green: G. sp1. Numbered icons in the **C**,**D** subfigures are identical with the populations sorted on Table 1 and Figure 1. For clarity only the group centroids are shown, with vertical and horizontal whiskers indicating the standard deviation of individual data. The explained variance in each axis is indicated in parenthesis. The original PCA showing all individuals data are available in Figure S2.

These analyses were performed only on those 60 individuals which were prepared by methods 1 and 2. Based on brain and muscle spectral analysis, the analytical method separated the Southern haplotype from the *G. obtusirostris* and G. sp1 by the first PC axis (Figure 3A,B). These findings were reinforced by the result of KW tests (Table 4). The confusion matrices showed the mean percentage of correctly classified cases was 73% for brain (range: 14/62%/and 22/87%/), 88% for the muscle (range: 14/54%/and 30/100%/) (Table 5). In both cases, high percentages of the samples were exchanged between the Southern and *G. obtusirostris* groups. While the G. sp1 group considerably separated from the other two, showing only three misclassified cases. In case of muscle samples almost half of the Southern individuals were reclassified into the *G. obtusirostris*. Otherwise the *G. obtusirostris* and G. sp1 groups showed almost complete isolation.

### 2.4. Population Level Identification

In the case of brain samples, only the Pop1 showed considerable separation from the other four along the PC1 axis, while on the second PC axis the Pop4 and 5 separated from the others significantly (Figure 3C,D and Table 6). In the case of muscle samples, all but one pairwise comparison was found to be significant in the PC1 axis. At the same time four from the ten pairwise comparisons were significant (see: Figure 3C,D and Table 6). The percentage of the correctly classified cases on population level showed high variability. Although, the mean percentage of correctly classified cases was 74%, and 70% for brain and muscle samples, respectively, the values ranged between 8 and 20 (42–83%) and 7 and 23 (33–92%) (Table 7).

## 3. Discussion

In the present work, the sensitivity of mass spectrometry for separating a cryptic freshwater fish complex was tested. Moreover, some additional analyses were made to clarify certain methodological issues, which can help improve the application of MALDI-TOF MS method in fish biology.

### 3.1. Effect of Sample Processing on the Result of MS Analyses

Although Nebbak and coworkers [46] stated that of the sample preservation methods, freezing has the least effect on MALDI-TOF MS results, our results suggest that the middle term sample storage, or the delayed sample preparation, fundamentally changed the results of MS analyses. Therefore, efforts should be made to process samples as quickly as possible. Moreover, this feature must be taken into consideration when evaluating and interpreting the results. Notwithstanding this, the delayed sample preparation appears to fundamentally affect the results of MS analysis; we did not find a single peak in the spectra, which would be specific/unique to this group. At the same time, several peaks were more frequent in the samples prepared after the 30 day incubation (Table S1). Using appropriate multivariate statistical methods, these frequent peaks are also appropriate to reliably separate these groups.

Our other methodologic question was to specify the possible effect of clove oil anesthesia on the results of MS analysis. This feature may have specific importance because clove oil is often used for anesthesia in fish biology [48,49,50]. The clove oil is used with preference because its active agent is eugenol (4-allyl-2-ethoxyphenol), which is a non-carcinogenic, non-mutagenic, and an “eco-friendly” substance [51,52]. It is also a safe material for the researcher, while having reduced negative physiological effects on fish compared with other narcotic agents [53]. Since no considerable effect was detected on the results of MS investigations, it seems that clove oil is a reliable anaesthetic for sample collection for mass spectrometry measurements.

### 3.2. MALDI-TOF MS Usability of Cryptic Species and Population Differentiation

The results of the PCA analysis, performed on MALDI-TOF MS data, correspond only partially with the results of the genetic study, but the results of the classification correspond well. Therefore, the MALDI-TOF MS can be used to reveal cryptic entities and populations as well (Figure 3). The relatively lower number of correctly classified cryptic species cases using brain samples is due to the fact that many individuals were exchanged between the Southern haplogroup and *G. obtusirostris*.

The results of the MS analysis of muscle and brain samples show highly similar results at the cryptic species level, but there are many differences in the results of analyses conducted on the population level. Moreover, the many misclassified cases suggest that the applicability limit of this method is being approached here.

In this work, no single unique peak was found to be characteristic for each population. Similarly to the results of methodologic surveys, only the differential distribution of peaks intensities can help to separate the individual populations in conjunction with multivariate statistical analysis. This is not altogether surprising due to the relatively few genetic differences, and the relatively low number of investigated samples per site (11–12 individuals per population). Additionally, the uneven distribution of sexes in each population may also have had a significant effect on the results. However, evaluating the effect of sexes on the results of rapid MSPP is beyond the scope of this work.

Our findings partly contradict the results of other researchers who have successfully used the MALDI-TOF MS to isolate molluscs and insect stocks [15,43]. In these cases, the method was used to separate two populations only. Whereas in our case, the MSPP was employed to classify the individuals into the five populations. Moreover, their investigations covered greater spatial scale and/or presumably larger genetic differences facilitating the separability of their samples. Additionally, in our case the studied species complex showed much more difficult phylogenetic features.

Vega Rúa and coworkers [16] stated that different body parts of arthropods may equally be used for the MALDI-TOF MS analysis. Similarly, our investigations showed both the muscle and brain samples produced usable MS profiles. The proportion of correctly classified cases was equal, or even higher among the muscle than in the brain tissue type (Table 3; Table 4). Therefore, it seems that muscle tissue is somewhat more appropriate for determining the intraspecific variability than brain samples. Additionally, this tissue type is much easier to be sampled by a biopsy in a living animal, therefore the tested individuals do not have to be terminated.

Due to our results, the application of MALDI-TOF MS technique could enter the service of phylogenetic and taxonomic research as a fast and cheap alternative of genetic studies to check the origin of stocked, economically important fish species. Additionally, this relatively fast and easily executable method is also be potentially usable for selecting breeding lineages in aquaculture projects. At the same time, the method limitations will need be taken into account in the cases where low number of individuals, or closely related stocks (populations) are compared.

## 4. Materials and Methods

### 4.1. Taxonomic Features of the Hungarian Stream Dwelling Gudgeons

Until the end of the 20th century, European gudgeon [*Gobio gobio* (Linnaeus, 1758)], a small-bodied cyprinid fish, was known as the only stream dwelling gudgeon species in Europe [54]. It was regarded as a wide-ranged super-species in western Eurasia, with many lotic, lentic, and intermediate forms. The European gudgeon was considered a common species in the Carpathian Basin as well, where it was noted as an indicator fish species of hilly streams [55]. The results of novel investigations [56,57] altered the taxonomy of this group. From the Carpathian basin, five genetically distinct groups were discovered [47,57,58]. From the valid species the *Gobio carpathicus* Vladykov, 1925, *G. gobio* are sporadic in Hungarian streams, the *Gobio obtusirostris* Cuvier and Valenciennes, 1842 is the dominant gudgeon species in the NW region of the Carpathian basin. In the SW area of the basin, and in the drainage system of Tisza River (eastern part of the basin) two, allopatric “cryptic” entities) are the dominant *Gobio* taxa. The taxonomic position of these two later mentioned haplogroups—the Southern and G. sp1—is still not clear. The above-mentioned features made this species complex suitable to be the target of our study. For more details see: Takács et al. 2014 [47].

### 4.2. Field, Sampling, and Preparation

Carpathian stream dwelling gudgeons (*Gobio* sp. *n* = 90) were used to the study. 18 individuals were collected (collection permits: PE-KTF/659-15/2017) from each of the five streams located to different areas of Hungary by electrofishing in the spring of 2017 (Figure 1A, and Table 1). Then fin clips were sampled for genetic investigations and stored in 96% ethanol at −20 °C until DNA extraction. To test the effect of sample procession methods on the result of MALDI-TOF MS analyses, three different sample preparation protocols were applied; (1) six from the collected 18 individuals per site were terminated by decapitation; (2) six additional individuals per site were euthanized by a lethal dose of clove oil and then decapitated. In the first two cases, after termination the whole brains and ~1 g skeletal muscles were dissected and kept at −80 °C until sample preparation. (3) The final six individuals were terminated by freezing whole animals. And in this case the sample preparation (whole brains and ~1 g skeletal muscles were dissected and kept at −80 °C) was made after 30 days of incubation at −30 °C.

### 4.3. Genetic Methods

Fin clips of the collected gudgeon specimens were sampled and stored in 96% ethanol at −20 °C until DNA sample collection. DNA was isolated with a DNeasy Blood and Tissue kit (Qiagen, Hilden, Germany), using 10–20 mg of fin tissue per the manufacturer’s instructions. Quality and quantity of the extracted DNA were checked using a NanoDrop 2000 c Spectrophotometer (Thermo Scientific, Waltham, MA, USA).

DNA of the 90 individuals was used for the amplification of the mitochondrial control region (mtCR). The sequences of mtCR were amplified by polymerase chain reaction (PCR) using the primers CR159 (CCCAAAGCAAGTACTAACGTC) and CR851 (TGCGATGGCTAACTCATAC) [57]. PCR was carried out using 0.2 mL of 5 U/mL Taq DNA polymerase (Fermentas), 2.5 mL of 10× Taq buffer, 1.7 mL MgCl2 (25 mM), 0.2 mL dNTPs (10 mM), 0.3 mL of each primer (20 mM), 2.0 mL template DNA, and 17.8 mL purified and distilled water in a final volume of 25 mL. The reactions were performed in a MJ Research PTC-200 Peltier Thermal Cycler under the following conditions: 95 °C for 1 min, followed by 37 cycles of 94 °C for 45 s, annealing at 52 °C for 30 s, and an extension temperature of 72 °C for 45 s, followed by a final extension at 72 °C for 8 min. PCR products were purified using the NucleoSpin^®^ Gel (Düren, Germany) and PCR Clean-up (Macherey Nagel GmbH, Düren, Germany) extraction kit. The subsequent determination of the nucleotide sequence of the PCR amplicons were performed using nucleotide sequencing by capillary electrophoresis (ABI 3130 Genetic Analyzer Device, ABI, Crosswall, London). This method applied bidirectional sequencing with the BigDye Terminator v3.1 Cycle Sequencing Kit, Performance Optimal Polimer 7 (ABI, Crosswall, London) and 50 cm capillary array according the recommendation of the producer. Sequences were trimmed manually using FinchTV 1.4.0 (Geospiza Inc, Seattle, WA, USA) and aligned using the ClustalX 2.0.11 software (Conway Institute UCD, Dublin, Ireland) [59]. Calculation of sequence polymorphism and haplotype detachment was performed using FaBox online software (Aarhus University, Aarhus, Denmark) [60]. The obtained sequences were compared with the ones uploaded to the GenBank using Blast online software (U.S. National Library of Medicine, Bethesda, MD, USA) [61]. Sequence divergence was calculated with net nucleotide substitution in MEGA5 [62], and a tree was constructed with the Maximum likelihood method using 2000 as the bootstrap value. A network was constructed using the median-joining algorithm in Network v. 4.6. [63] software (Fluxus Technology Ltd., Colchester, England). Similar haplotypes were classified arbitrarily into haplogroups (see ‘‘enframings’’ in Figure 1C–E).

### 4.4. Sample Preparation, Proteomic Analysis and Data Processing for MALDI-TOF MS

Frozen fish tissues were homogenized using a TissueLyser LT (QIAGEN) after the addition of 300 µL/g extraction solution (50% acetonitrile and 2.5% aqueous trifluoro-acetic acid) and were prepared with a high-energy ultrasonicator UI250 V (Hielsher Ultrasound Technology, Teltow, Germany) for 6 × 10 s, applying ice-cooling between cycles. The homogenates were then vortexed and centrifuged (Heraeus Biofuge Pico, Thermo Fisher Scientific, Waltham, MA, USA) at 10,000 rpm for 5 min. The clear supernatant was moved into a new sample tube. One μL of each supernatant protein sample was deposited onto a 96-position MALDI-TOF target plate (Bruker Daltonics, Bremen, Germany) in three replicates, allowed to dry at room temperature, and overlaid with 1 μL of the matrix solution, containing saturated α-cyano-4-hydroxycin-namic acid (α-CHCA) (Sigma-Aldrich, St Louis, MO, USA) in 50% acetonitrile and 2.5% aqueous trifluoro-acetic acid (TFA). The matrix sample spots were crystallized by air drying. After drying, the plate was inserted into the instrument for MALDI-TOF MS analysis.

MALDI-TOF measurements were carried out using a MALDI Microflex LT (Bruker Daltonics, Bremen, Germany), equipped with a nitrogen laser (337 nm). Mass spectra were acquired using the Flex Control 3.0 software (Bruker Daltonics, Bremen, Germany) in automatic and linear mode within a mass range between 2 and 20 kDa. Each spectrum was collected in the positive ion mode after an average of 240 laser shots. Bacterial Test Standard (Bruker Daltonics, Bremen, Germany) was used for calibration of the instrument. One spectrum per sample (*n* = 3 replicates) was obtained to assess the suitability of mass spectrometric approach for identification of cryptic species. Mass data files were then exported from the FlexAnalysis 3.0 software (Bruker Daltonics, Bremen, Germany) and transformed to mzXML-files (*m*/*z*-intensity lists). The mzXML files were then imported in the free statistical software Mass-Up (Mass-Up, Vigo, Spain), for management of MALDI-TOF mass spectra data [64,65]. This software allows the detection of potential biomarkers, enabling the construction of models for automatic classification based on differences in the mass spectra. Data processing and analyses were executed following the suggestions of Fernandez-Alvarez and coworkers, and Yolanda and coworkers [65,66]. Therefore, each spectrum was smoothed by the moving average method, baseline corrected by the top hat method, and peak detection was carried out using the MassSpecWavelet method with a signal-to-noise ratio 6. The spectra from fish belonging to different cryptic species were compared using the forward method to obtain inter-sample matching, requiring a peak match score of 300 ppm. Result reports, which characterize the accuracy of the classification procedure executed on a population, cryptic species and preparation type levels are available in Table S2. Confusion matrices were computed using NaiveBayes model. Principal component analysis (PCA) was performed using intensity values of the detected peaks converted into binary file by Past 2.12 statistical software [67]. For the clarity and visibility of our results only the group centroids and the standard deviations of the individual PC1 and PC2 coordinates were indicated. To characterise the group separations the PCA plot coordinates of the studied individuals were revealed using non-parametric pairwise Kruskal-Wallis (KW) tests in all cases.

## Figures and Tables

**Table 1:** Stream codes, names, sampling sites, coordinates, collection dates, and the number of individuals showing the same haplotype. ∑^1^: number of individuals per site ∑^2^: number of individuals classified into a certain haplotype.

N^o^	Stream Name	Sample Site	Coordinate	Collection Date	G. sp1			*G. obtusirostris*		Southern h.		∑^1^
					H01	H02	H03	H04	H05	H06	H07	
Pop1	Tolcsva	Erdőhorváti	N48.31088, E21.43026	2017.03.24	13	3	2					18
Pop2	Csernely	Uppony	N48.21437, E20.44003	2017.03.24	13	5						18
Pop3	Kemence	Bernecebaráti	N48.04741, E18.91824	2017.03.25				17	1			18
Pop4	Tapolca	Raposka	N46.85051, E17.42178	2017.03.31				1		16	1	18
Pop5	Egervíz	Gyulakeszi	N46.87300, E17.47000	2017.05.02				14		4		18
				∑^2^	26	8	2	32	1	20	1	90

**Table 2:** Results of Kruskal-Wallis pairwise comparisons of the three sample preparation types’ PCA data. The significant pairwise group differences/*p* < 0.05/are italicized.

Tissue Type	Method	PC1 Axis			PC2 Axis		
		Prep. Type 1	Prep. Type 2	Prep. Type 3	Prep. Type 1	Prep. Type 2	Prep. Type 3
**Brain**	**Prep. Type 1**	-			-		
	**Prep. Type 2**	0.0958	-		*0.0028*	-	
	**Prep. Type 3**	*<0.0001*	*<0.0001*	-	0.3890	0.0575	-
**Muscle**	**Prep. Type 1**	-			-		
	**Prep. Type 2**	1.0000	-		*<0.0001*	*-*	
	**Prep. Type 3**	*<0.0001*	*<0.0001*	-	*0.0022*	*0.0126*	-

**Table 3:** Classification results of the spectral datasets derived from brain and muscle tissue samples prepared by three different methodologies. Prep. type 1: immediate preparation, Prep. type 2: clove oil euthanization and immediate preparation. Prep. type 3: sample preparation in the lab after 30 days incubation at −30 °C. In the confusion matrices the actual and predicted groups are presented in rows and in columns, respectively. Both the numbers and the percentage of the cases are indicated. The correctly classified cases (CCC) are indicated by bold letter type and underlined in the diagonals of the tables. The overall CCC is the ratio of correctly classified individuals to the total number of individuals. ∑: number of individuals originally classified into a certain group. PGM: predicted group membership. *: in the second group one individual’s brain sample MS analysis was failed.

Tissue Type	Brain					Muscle				
**Method**	**Prep. Type 1**	**Prep. Type 2**	**Prep. Type 3**	**∑**	**Overall CCC**	**Prep. Type 1**	**Prep. Type 2**	**Prep. Type 3**	**∑**	**Overall CCC**
**Prep. Type 1**	**17**	13	0	30	**53 (59.6%)**	**16**	12	2	30	**60 (66.6%)**
**Prep. Type 2**	13	**14**	2	29 *		13	**14**	3	30	
**Prep. Type 3**	3	5	**22**	30		0	0	**30**	30	
**PGM**	33	32	24	**89**		29	26	35	**90**	

**Table 4:** Group differentiations of the three cryptic species based on pairwise KW tests. Significant group differences/*p* < 0.05/are italicized.

Tissue Type		PC1 Axis			PC2 Axis		
		Southern h.	*G. obt*.	G. sp1	Southern h.	*G. obt*.	G. sp1
**Brain**	**Southern h.**	-			-		
	***G. obt.***	*0.0003*	-		1.0000	-	
	**G. sp1**	*0.0066*	1.0000	-	0.6044	*0.0319*	-
**Muscle**	**Southern h.**	-			-		
	***G. obt.***	*0.0002*	-		0.4427	-	
	**G. sp1**	*0.0002*	1.0000	-	1.0000	0.0578	-

**Table 5:** Classification results of the genetically identified individuals sorted into the three cryptic species (haplogroups) by MSPP. In the confusion matrices the actual and predicted groups are presented in rows, and in columns, respectively. The correctly classified cases (CCC) are indicated by bold letter type in the diagonals of the tables and underlined. The overall CCC is the ratio of correctly classified individuals to the total number of individuals. ∑: number of individuals originally classified into a certain group. PGM: predicted group membership.

Tissue Type	Group	Southern h.	*G. obt.*	G. sp1	Σ	Overall CCC
**Brain**	**Southern h.**	**8**	3	2	13	**43 (73%)**
	***G. obt.***	4	**15**	4	23	
	**G. sp1**	0	3	**20**	23	
	**PGM**	12	21	26	59	
**Muscle**	**Southern h.**	**7**	6	0	13	**53 (88%)**
	***G. obt.***	0	**23**	0	23	
	**G. sp1**	0	1	**23**	24	
	**PGM**	7	30	23	60	

**Table 6:** Group differentiations of the five surveyed populations based on the results of pairwise KW tests. Significant group differences/*p* < 0.05/are italicized.

Tissue Type		PC1 Axis					PC2 Axis				
		Pop1	Pop2	Pop3	Pop4	Pop5	Pop1	Pop2	Pop3	Pop4	Pop5
**Brain**	**Pop1**	-					-				
	**Pop2**	*0.0123*	-				1.0000	-			
	**Pop3**	0.4547	0.2623	-			1.0000	1.0000	-		
	**Pop4**	*0.0099*	1.0000	0.1937	-		*0.0025*	*0.0031*	*0.0012*	*-*	
	**Pop5**	*0.0099*	1.0000	0.3038	1.0000	-	*0.0188*	*0.0135*	*0.0244*	*0.0005*	-
**Muscle**	**Pop1**	-					-				
	**Pop2**	*0.0005*	-				*0.0031*	-			
	**Pop3**	*0.0201*	*0.0111*	-			*0.0016*	1.0000	-		
	**Pop4**	*0.0004*	*0.0012*	*0.0004*	-		1.0000	*0.0135*	*0.0020*	-	
	**Pop5**	*0.0006*	1.0000	*0.0031*	*0.0008*	-	*0.0012*	1.0000	1.0000	*0.0031*	-

**Table 7:** Results of population level classifications using MSPP. In the confusion matrix the actual and predicted groups are presented in rows and in columns, respectively. The correctly classified cases (CCC) are indicated by bold letter type and underlined. ∑: number of individuals per site.

Sample	Group	Pop1	Pop2	Pop3	Pop4	Pop5	Σ	Overall CCC
**Brain**	**Pop1**	**10**	0	1	0	0	11	**44 (74%)**
	**Pop2**	1	**9**	2	0	0	12	
	**Pop3**	2	1	**5**	3	1	12	
	**Pop4**	1	0	0	**10**	1	12	
	**Pop5**	0	0	0	2	**10**	12	
	**PGM**	14	10	8	15	12	**59**	
**Muscle**	**Pop1**	**9**	0	2	0	1	12	**42 (70%)**
	**Pop2**	1	**11**	0	0	0	12	
	**Pop3**	4	1	**4**	1	2	12	
	**Pop4**	2	0	1	**8**	1	12	
	**Pop5**	0	0	1	1	**10**	12	
	**PGM**	16	12	8	10	14	**60**	

## Supplementary Materials

The following are available online, Table S1: Details of the detected peaks and results of statistical analyses. Table S2: Result reports of Mass-Up software analyses. Figures S1 and S2: Principal Component Analysis plots of MALDI-TOF MS spectral datasets derived from gudgeon brain and muscle tissues, Figure S3: representative mass spectra of brain and muscle samples.
