# Supplementary material for: Testing the Applicability of MALDI-TOF MS as an Alternative Stock Identification Method in a Cryptic Species Complex
Source: Molecules. 2020 Jul 14;25(14):3214. doi: 10.3390/molecules25143214 (PMC7397217; doi:10.3390/molecules25143214)
Supplement: Supplementary file 1 [file molecules-25-03214-s001.zip › Supplementary Figures.docx]

**Supplementary Figures**


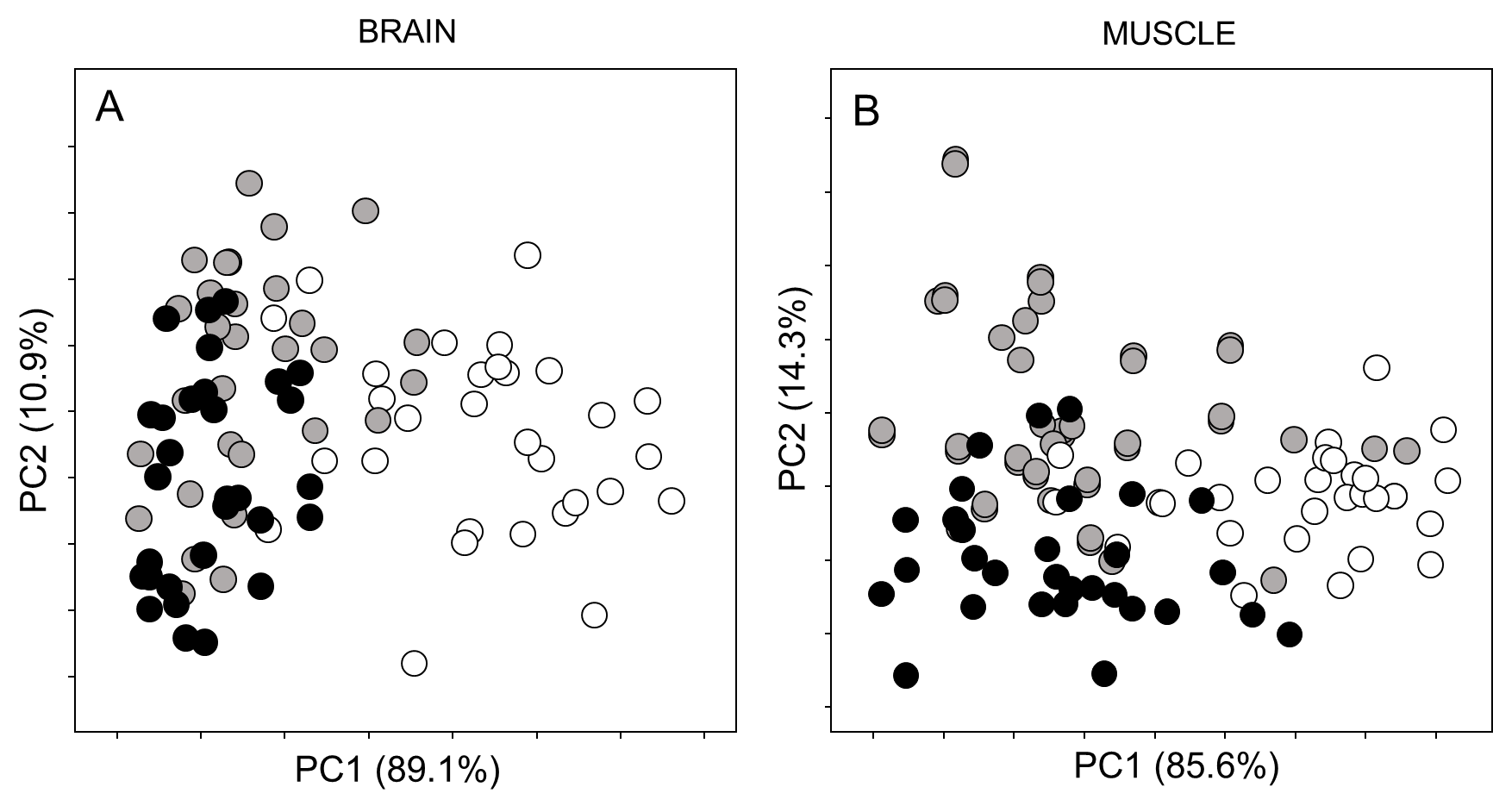


**Figure S1.** Principal Component Analysis plots of MALDI-TOF MS spectral datasets derived from gudgeon brain (A) and muscle (B) tissues. Black (Prep. Type 1): immediate field prepared samples; Grey (Prep. Type 2): clove oil euthanization + immediate field prepared samples; White (Prep. Type 3): the whole body was frozen and prepared in the lab after 30 days incubation on -30°C. The explained variance in each axis is indicated in parenthesis.


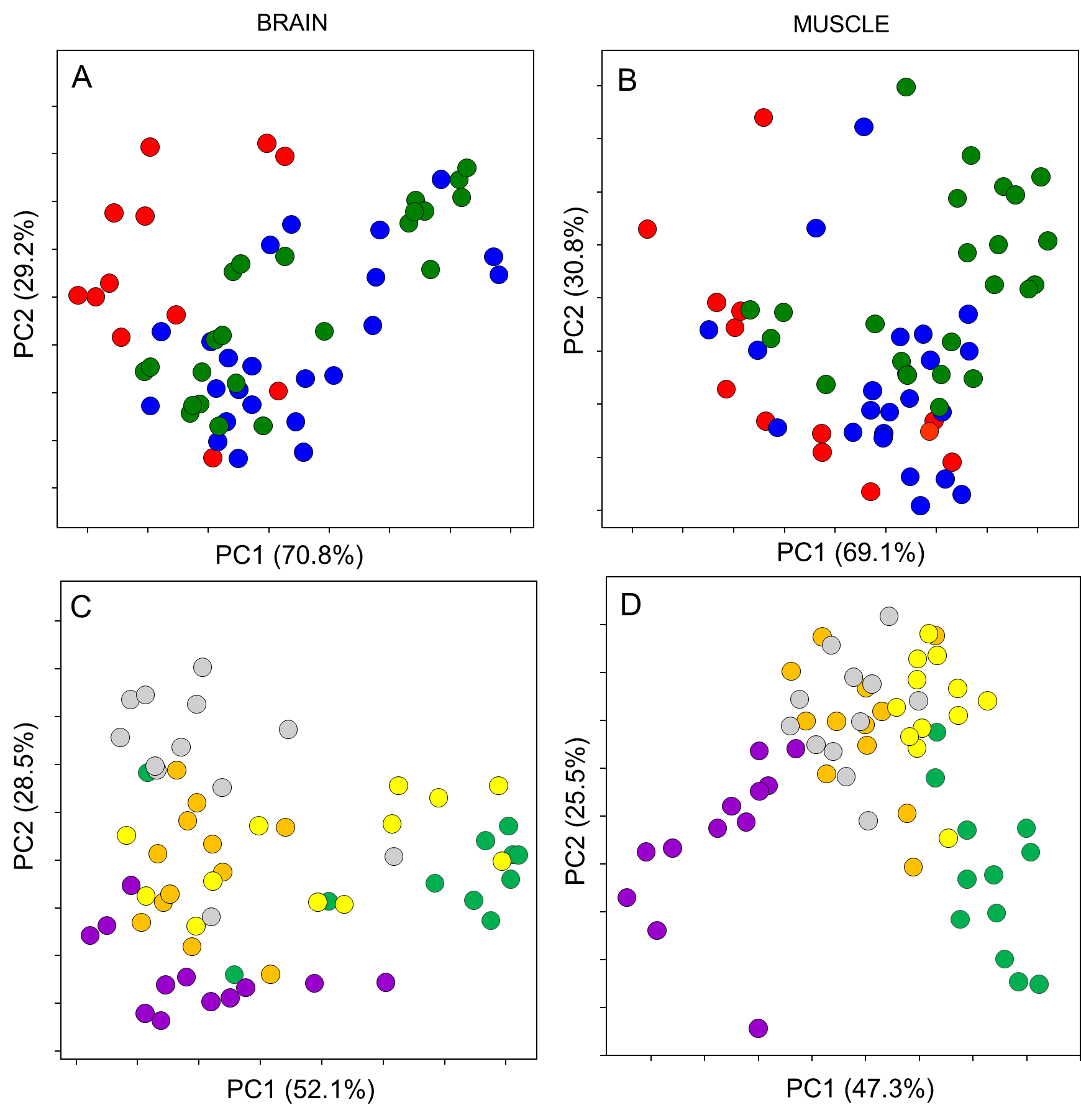


**Figure S2** Principal Component Analysis plots of MALDI-TOF MS spectral datasets derived from gudgeon brain (A) and muscle (B) tissues. Groups were set up by genetic features (A-B) and collection site (C-D). On subfigures A and B: Red circle: “Southern haplogroup”, Blue: *G. obtusirostris*, Green: G. sp1. On subfigures C and D: Light green: Pop1, Orange: Pop2, Yellow: Pop3, Purple: Pop4, light grey: Pop5. The explained variance in each axis is indicated in parenthesis


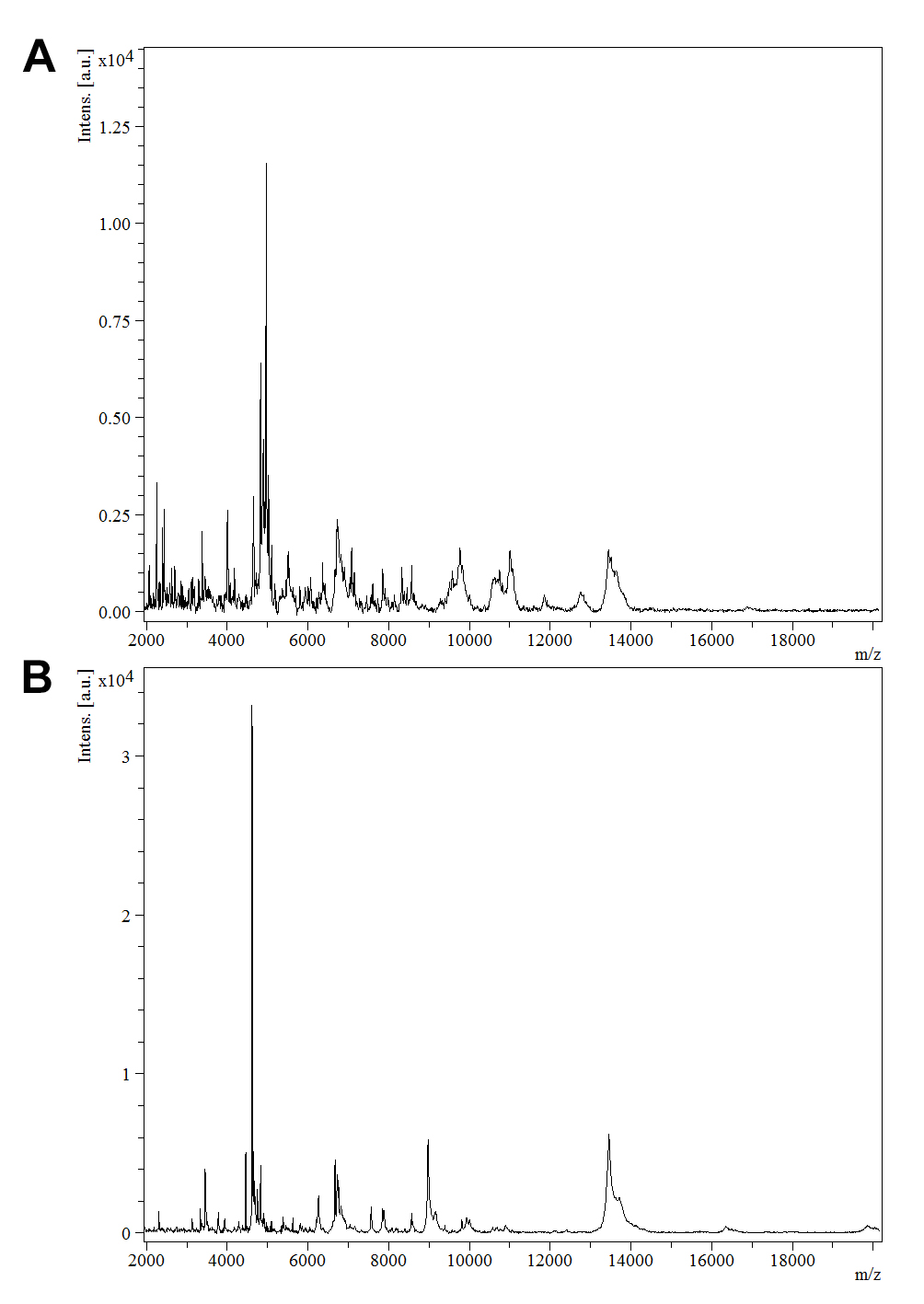


**Figure S3** Representative matrix-assisted laser desorption/ionization time-of-flight mass spectrometry (MALDI-TOF MS) spectra of brain (A) and muscle (B) samples. Mass spectra were visualized by FlexAnalysis 3.0 software (Bruker Daltonics, Bremen, Germany) within an identical range from 2,000 to 20,000 m/z. Mass spectra were smoothed with the Savitzky-Golay method and the baseline was removed based on TopHat algorithm. a.u., arbitrary units; m/z, mass-to-charge ratio
